# Implementing a well-being curriculum in anesthesiology residency: insights from a teaching hospital in Brazil

**DOI:** 10.1016/j.bjane.2025.844601

**Published:** 2025-02-22

**Authors:** Claudia M.R.P. Cavaliere, Lorena I.M. Carvalho, Liana M.T.A. Azi, Edgar Yugue, Renata de Paula Lian, Marcos A.C. Albuquerque

**Affiliations:** a Pontifícia Universidade Católica de Campinas (PUC-Campinas), Departamento de Anestesiologia, Campinas, SP, Brazil; b Hospital Unimed Primavera, Departamento de Anestesiologia, Teresina, PI, Brazil; c Universidade Federal da Bahia, Departamento de Anestesiologia, Salvador, BA, Brazil; d Universidade Federal de Sergipe, Departamento de Anestesiologia, Aracaju, SE, Brazil

Dear Editor,

Medical residency is a critical phase in the career development of physicians. Nonetheless, it is also a period marked by significant transitions and changes, often leading to increased stress and a higher risk of Burnout Syndrome. Burnout Syndrome is characterized as a state of work-induced exhaustion that comprises three main elements: 1) Emotional Exhaustion (EE), 2) Depersonalization (DP), and 3) Reduced Personal Accomplishment (PA), affecting the sense of fulfillment and performance.^1^

Burnout has a major impact on patient care, resulting in poor outcomes, medication errors, and compromised safety. For healthcare providers, it can lead to cardiovascular diseases, substance abuse, depression, and risk of suicide.^2^ Importantly, suicide has been pointed as the second leading cause of death among Medical Residents (MRs). Key factors contributing to burnout include long working hours, lack of rest, insufficient sleep, high academic pressures, and the difficulties in managing patient care.^3^

Anesthesiology residency programs typically emphasize the development of technical skills for clinical practice, minimizing non-technical areas such as resilience, communication, and mindfulness.^2^, ^3^, ^4^ Thus, wellness strategies for resident physicians remain largely individual efforts, lacking attention in official medical residency programs.

This project aims to present the experience of implementing a well-being curriculum program for Anesthesiology medical residency, designed to mitigate the impact of work-related stressors and foster a culture of self-care within the field of Anesthesiology.

A pilot project named the Well-being Medical Residency Program (WMRP) was initiated in April 2022 at the Department of Anesthesiology, Pontifícia Universidade Católica de Campinas (PUC-Campinas) teaching Hospital, São Paulo, Brazil. The Anesthesiology residency program at PUC-Campinas, affiliated with the Brazilian Society of Anesthesiology (SBA), includes 31 staff anesthesiologists and 24 MRs, with 8 MRs per year over a total three-year training period (MR-1, MR-2 and MR-3). The program received comprehensive approval from the institutional and clinical boards: the Medical Residency Commitee (COREME), the Anesthesiology Department Supervisor, and SBA.

The curriculum was developed based on previous wellness programs for Anesthesiology MRs,^5^^,^^6^ and incorporated burnout interventions^7^ aligned with the local cultural, human, and financial resources. All MRs and anesthesiology staff were invited to join, engaging in the program upon personal consent. A WMRP Committee was established to plan, organize and supervise the program. A Medical Residents Committee was formed for communication and representation of the MRs.

A modular curriculum was developed to address eight key areas, implemented throughout the academic year: 1) Physician wellness; 2) Physician resilience; 3) Professionalism; 4) Occupational well-being; 5) Emotional well-being; 6) Financial and career management; 7) Social well-being and teamwork; and 8) Situational conscience and mindfulness.

The WMRP design included multiple interventions to foster self-care and self-awareness among MRs. Organizational interventions: 1) Monthly WMRP Committee and MR Committee meetings; and 2) Online classes, group discussions, live testimonials, and guided conversations. Individual interventions: 1) Encouragement to seek professional mental health support; 2) A mentorship program; 3) Social activities: happy hours, gatherings and birthday celebrations, welcoming reception to new MRs, graduation ceremony and party. 4) Decompression social area inside the hospital. 5) Encouragement of balanced eating, exercise, and physical evaluations; 6) Access to medical and dental care; 7) Mental health support (provided by COREME).

The Maslach Burnout Inventory (MBI) scale was administered anonymously to all participants in the program. The thresholds for each category were: EE ≥ 27, DP ≥ 10, and PA ≤ 33. High burnout risk was indicated by a score of EE ≥ 27 and/or DP ≥10,^8^ and the number of categories per individual was evaluated. Qualitative evaluation and a satisfaction questionnaire were also administered.

Data were analyzed using descriptive statistics in IBM SPSS Statistics version 30.0. The Chi-Square test was employed for non-parametric independent variables, while the *t*-test was used for parametric variables. A p-value of less than 0.05 was considered statistically significant.

The WMRP was officially launched on June 27, 2022, with a meeting at the Radisson Red Campinas Hotel, attended by 52 participants. Program adherence improved over time, supported by a WhatsApp group for internal communication. By September 2022, 49 individuals had agreed to participate, including 29 out of 31 staff anesthesiologists, all 8 MR-1, 7 out of 8 MR-2, and 5 out of 8 MR-3. Eventually, the remaining 2 staff and 1 MR-2 joined, bringing the total to 52 participants.

From September 2022 to February 2023, four online classes were held, but these were later discontinued due to low participation. The WMRP Committee was established in September 2022 and held monthly meetings, comprising 5 staff anesthesiologists, along with a MRs Committee of 5 MRs. Social gatherings, such as birthday celebrations (held six times) and happy hours (held twice), were later discontinued due to budget constraints.

In September 2022, the MBI scale was administered anonymously, with 19 staff and 25 MRs responding, indicating a high burnout risk both in 57.9% of staff and 80% of MRs (Table 1). In the analysis of the MBI categories among participants, the MRs showed a significantly higher likelihood of experiencing combinations of either EE with DP, EE with PA, or DP with PA compared to the staff (Fig. 1).

**Table 1:** Maslach Burnout Inventory scale results for participants of the Well-being Medical Residency Program (staff and medical residents).

MBI category	Staff					MRs					p-value
	n (%)					n (%)					
	Yes		No		Total	Yes		No		Total	
EE	10	(52.6%)	9	(47.4%)	19	20	(80%)	5	(20%)	25	0.05
DP	6	(31.6%)	13	(68.4%)	19	9	(36%)	16	(64%)	25	0.75
PA	4	(21.1%)	15	(78.9%)	19	9	(36%)	16	(64%)	25	0.28
High burnout risk	11	(57.9%)	8	(42.1%)	19	20	(80%)	5	(20%)	25	0.11

MBI, Malash Burnout Inventory; EE, Emotional Exhaustion (≥ 27); DP, Depersonalization (≥ 10); PA, Low Personal Accomplishment (≤ 33); MRs, Medical Residents.

High burnout risk = presence of high levels of EE (≥ 27) and/or DP (≥ 10).

**Figure 1:**
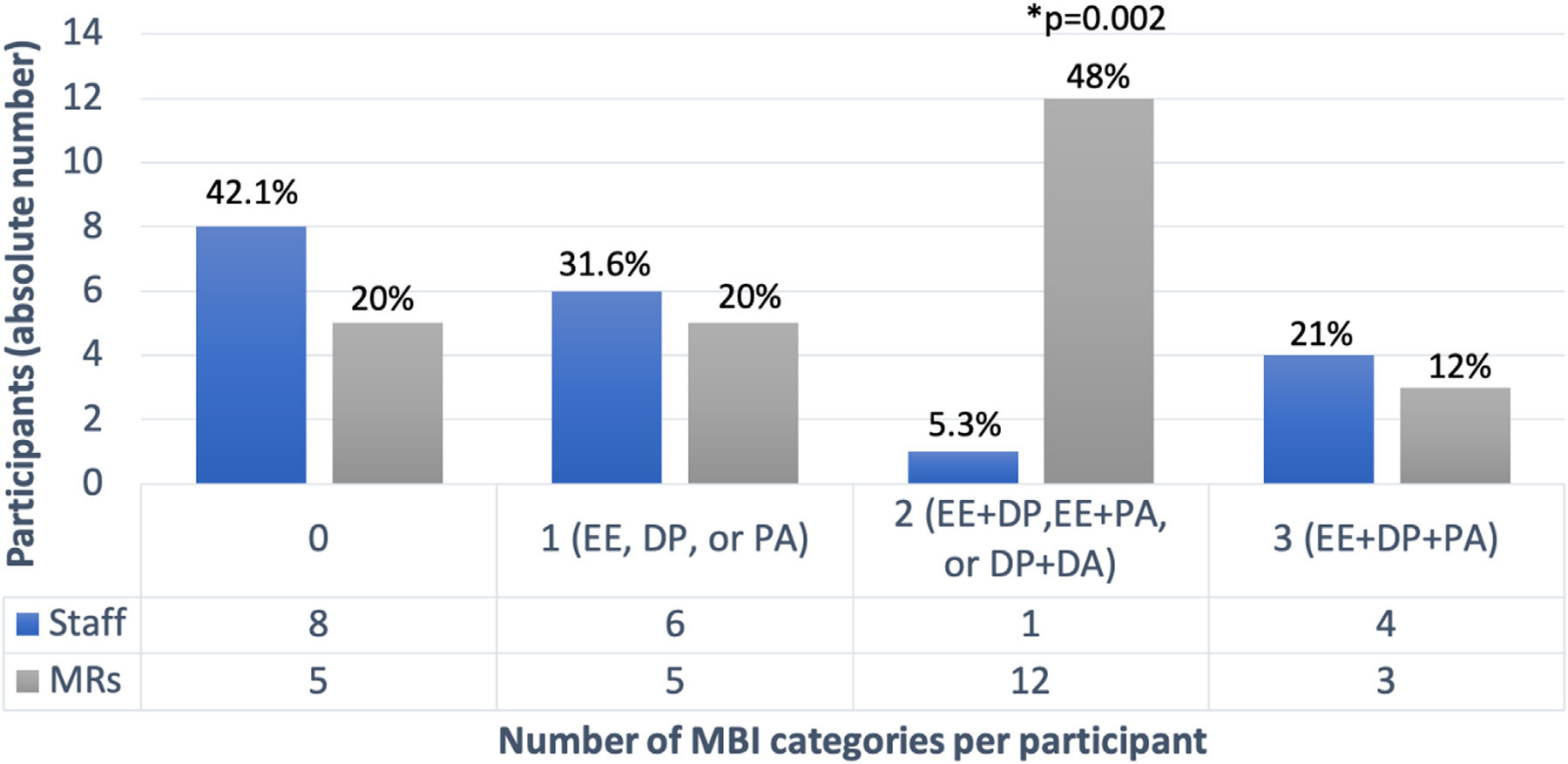
Number of Maslach Burnout Inventory (MBI) categories per individual and increasing burnout risk in participants of the Well-being Medical Residency Program (staff and medical residents). MRs, Medical Residents; MBI, Maslach Burnout Inventory; EE, Emotional Exhaustion; DP, Depersonalization; PA, Personal Accomplishment (*p < 0.05).

A satisfaction questionnaire conducted in April 2023 received 46 responses (50% MRs and 50% staff), with 91.3% indicating that social events, similar to the graduation ceremony, could enhance community and institution well-being. Regarding the perception of the WMRP, all participants expressed belief in the value of the project and emphasized the importance of maintaining the initiative.

Major challenges identified by the WMRP Committee included: initiating the WMRP in the middle of the academic year, insufficient financial resources, initial resistance from staff and MRs to participate, lack of prior similar projects for reference, and difficulty organizing social events due to demanding work schedules.

The WMRP demonstrated significant engagement from MRs and staff in the Department of Anesthesiology at PUC-Campinas and highlighted critical areas for improvement in well-being. Initial findings revealed high levels of EE and a high burnout risk, underscoring the necessity of sustained wellness initiatives.

Previous meta-analyses suggest that MRs in surgical specialties, including anesthesiology, have the highest prevalence of burnout syndrome among physicians, with rates reaching up to 50%, and depression rates ranging between 10% and 22%.^8^, ^9^, ^10^ The burnout prevalence observed in our WMRP was similar to these findings.

The high-stress and demanding work environment can significantly alter MR performance, potentially compromising patient care. Studies have shown that MRs experiencing burnout are twice as likely to be involved in patient safety incidents and provide suboptimal care. Burnout also affects professionalism and the overall work environment, leading to strained relationships with colleagues and patients.^9^ To mitigate these risks some studies suggest that residency programs should implement comprehensive wellness programs that include mental health support, resilience training, and efforts to improve work-life balance.^2^

The demanding nature of their work, which often includes shifts of 24 hours or more, contributes to significant sleep debt, which has been linked to decreased alertness, depression, fatigue and other health issues. Additionally, the high workload and intense working environment further exacerbate burnout and exhaustion, negatively impacting both residents’ health and the quality of patient care.^9^

Approaches to conceptualize and intervene in MR wellness include acknowledging and assessing the problem, harnessing the power of leadership, developing and implementing targeted interventions, cultivating community in the workplace, using rewards and incentives wisely, aligning values and strengthening culture, promoting flexibility and work-life integration, providing resources to promote resilience and self-care, and facilitating and funding organizational science.^2^^,^^3^ These strategies address multiple stressors through targeted interventions and supportive measures^4^ and were thoroughly evaluated by our modular curriculum.

Previous studies have largely focused on individual approaches for burnout. However, these isolated interventions are generally less effective compared to systemic changes, which more significantly impact the environment, leadership, and team communication.^3^ A key strength of our initiative is that it comprehensively integrates both individual and organizational strategies. We highlight the full institutional support and the active participation of staff and MRs, demonstrating a greater impact on embracing the curriculum.

Notably, over 90% of both residents and staff in our project viewed social gatherings as beneficial in enhancing relationships and well-being. This aligns with previous research on wellness curricula in anesthesiology, which found that participation in social events, such as dinners, was linked to lower DP scores on the MBI scale.^6^

The WMRP has several limitations. It was conducted in a single institution, limiting the results to that specific setting. Some initial resistance from MRs and staff led to delayed participation, possibly affecting their full potential response to the interventions. Additionally, classes and social gatherings were discontinued due to low attendance, organizational challenges, and insufficient funding. As for the MBI scale, the single moment measurement may not reflect individual responses in other time periods. Nevertheless, the major acknowledgement of the WMRP raises an urgent need for changes in traditional anesthesiology training and education.

In conclusion, implementing a well-being curriculum in anesthesiology residency training resulted in positive changes within our program. The promotion of a well-being culture was pursued through a combination of organizational and individual interventions targeting both residents and staff, offering an innovative educational approach in anesthesiology. Further research is needed to identify the most effective interventions in anesthesiology and to adapt validated programs to different contexts.

## Conflicts of interest

The authors declare no conflicts of interest.
